# Magnetocaloric Effect, Structure, Spinodal Decomposition and Phase Transformations Heusler Alloy Ni-Mn-In

**DOI:** 10.3390/nano13081385

**Published:** 2023-04-16

**Authors:** D. D. Kuznetsov, E. I. Kuznetsova, A. V. Mashirov, A. S. Loshachenko, D. V. Danilov, V. I. Mitsiuk, A. S. Kuznetsov, V. G. Shavrov, V. V. Koledov, P. Ari-Gur

**Affiliations:** 1 Kotelnikov Institute of Radioengineering and Electronics of Russian Academy of Sciences, 125009 Moscow, Russia; 2 M.N. Miheev Institute of Metal Physics of Ural Branch of Russian Academy of Sciences, 620108 Ekaterinburg, Russia; 3 Department of Physics, The Ural State Agrarian University, 620075 Yekaterinburg, Russia; 4 IRC Nanotechnology, Research Park, St. Petersburg State University, 199034 St. Petersburg, Russia; 5 Scientific-Practical Materials Research Center of National Academy of Sciences of Belarus, 220072 Minsk, Belarus; 6 Department of Mechanical and Aerospace Engineering, Western Michigan University, Kalamazoo, MI 49008, USA

**Keywords:** martensitic transformation, metamagnetostructural transformation, magnetocaloric effect, premartensitic states, nano austenite, nano martensite, spinodal decomposition, high resolution transmission electron microscopy (HR TEM), magnetization transmission, electron microscopy

## Abstract

Ni_46_Mn_41_In_13_ (close to 2-1-1 system) Heusler alloy was studied by magnetization measurement dependence on the temperature in magnetic fields of up to 13.5 T. The magnetocaloric effect measured by the direct method in quasi-adiabatic conditions showed a maximum value of ∆T_ad_ = −4.2 K at a temperature T = 212 K in a magnetic field of 10 T in the region of martensitic transformation. The structure of the alloy was studied by transmission electron microscopy (TEM) as a function of the temperature and the thickness of the sample foil. In the temperature range from 353 to 215 K, at least two processes were established. The results of the study indicate that the concentration stratification occurs according to the mechanism of spinodal decomposition (conditionally spinodal decomposition) into nanoscale regions. At a temperature of 215 K and lower, martensitic phase with 14 M modulation is observed in the alloy at thicknesses greater than 50 nm. Some austenite is also observed. In foils with thickness of less than 50 nm in a temperature range from 353 to 100 Km only the initial austenite, which has not transformed, was found.

## 1. Introduction

Heusler alloys of various systems, particularly those based on Ni-Mn-In, are promising materials with multifunctional properties for the future design of thermally and magnetically controlled micro- and nanoactuators and solid-state magnetic refrigerators. By varying both the chemical and phase composition, it is possible to change the characteristic temperatures of the metamagnetostructural transformation of thermoelastic martensitic type transformation, which greatly expands the possibilities of application. Therefore, the study of such materials, especially their crystalline structure, is necessary to achieve the maximum values of the beneficial effects and increase the strength and stability of materials during operation [1,2]. Heusler alloys represent a wide range of materials, which are commonly understood as chemical compounds with structural ordering, such as L2_1_, that transitions upon cooling to a low symmetry phase (martensite). So Heusler alloys Ni-Mn-In, both in stoichiometric and non-stoichiometric compositions, often demonstrate a sequence of structural and magnetic phase transformations whose temperatures can be carefully tailored giving rise to attractive multifunctional properties in a useful temperature range [1,2,3].

One of the important properties of considerable interest of researchers in Heusler alloys of the Ni-Mn-X family (X = In, Sn, Sb) is the magnetocaloric effect (MCE), which demonstrates high values in the region of martensitic transformation [4,5]. It is known [6] that the MCE is characterized by a change in temperature ∆T_ad_ under adiabatic conditions (direct measurements) and a change in entropy ∆S_mag_ in isothermal conditions (indirect measurements) at the application of an external magnetic field and can be both conventional and inverse. A certain composition of the Ni-Mn-X (X = In, Sn, Sb) alloys are shown to exhibit an inverse MCE in the vicinity of the structural transformation of martensitic phase to austenitic phase, while the alloys of the system Ni-Mn-Ga have a conventional MCE.

In general, as the temperature decreases the high-temperature austenitic state of the nonstoichiometric Heusler alloys Ni_x_Mn_y_In_z_ can undergo a series of disorder–order transitions A2→B2→L2_1_, depending on the occupancy of the positions of the X, Y, and Z sublattices [7]. At temperatures above 1220 K, an A2-type phase is observed, in which the atoms of the X, Y, and Z elements are randomly distributed over the sites of the crystal lattice. As the sample is cooled, the degree of disorder decreases and, upon cooling to 990 K, the alloy transforms into a B2-type phase, in which the atoms of element X occupy positions at the corners of the crystal lattice, and elements Y and Z occupy positions with equal probability at the center of the cubic cell. Upon further cooling, the alloy transforms from the partially ordered state B2 to the ordered phase L2_1_. Any deviation from this stoichiometry leads to decomposition, and the alloys prefer to have an energetically favorable structure with a 2-1-1 stoichiometry. The rest of the alloys can form different structures with different compositions.

The focus of the present study is on the structure of Heusler alloys of non-stoichiometric compositions, since it has not yet been shown reliably whether it is possible to stabilize in them a single-phase, austenitic L2_1_-type ordered phase. The B2→L2_1_ transformation in these compounds requires very long annealing at low temperatures, and diffusion processes proceed more slowly below 600 K; as a result, a certain amount of a disordered phase may be present at room temperature along with a highly ordered phase.

Work on the study of order–disorder transitions in alloys based on Heusler Ni–Mn–In showed that, for example, in Ni_45_Co_5_Mn_50−x_In_x_, the transformation temperature B2→L2_1_ is approximately 896 K [8,9] and the portion of the L2_1_ phase increases with cooling. This means that the structural order present at room temperature depends on the cooling conditions, in particular on the rate [10].

In addition, non-stoichiometric Heusler alloys with an excess of manganese have structural metastability, which manifests itself in the coexistence of two phases; namely, they transform into a composite made of L2_1_(Ni_50_Mn_25_X_25_) + L1_0_(Ni_50_Mn_50_) [11]. The decomposition process occurs during slow cooling or during annealing of ternary Heusler alloys below the B2→L2_1_ transformation temperature. In [12], it is shown that during the decomposition of the Ni_50_Mn_45_In_5_ alloy at 650, 700, and 750 K, inclusions of the L2_1_ phase with Ni_50_Mn_25_In_25_ composition are formed, embedded in a matrix of the L1_0_ phase with Ni_50_Mn_50_ composition. These phases are in different magnetic states: the inclusions are paramagnetic, whereas the matrix is antiferromagnetic. The interaction between them leads to a phenomenon called «shell ferromagnetism» [12].

This paper presents the study, by transmission electron microscopy (TEM), of the structural features of Ni_46_Mn_41_In_13_ nonstoichiometric Heusler alloy that affect the magnetostructural transformations in this alloy. The study of the thermoelastic metamagnetostructural transition in both bulk samples and nanoscale foils of this alloy, under high magnetic fields and temperature, is also an aim of the present work.

## 2. Experimental

Polycrystalline samples of the Ni_46_Mn_41_In_13_ alloy (e/a = 7.86) were arc melted and subsequently vacuum annealed, as described in the [13]. X-ray diffraction studies at room temperature were carried out using Cu K-alpha radiation on a Rigaku Smartlab X-ray diffractometer. The magnetization was measured by the induction method on the cryogenics setup in magnetic fields up to 13.5 T. Measurements of the magnetocaloric effect were made by the direct method in stationary magnetic fields 2, 5, and 10 T on the original setup described in [14]. Electron microscopy studies, including electron diffraction and TEM imaging in bright and dark fields, were carried out with an electron beam accelerating voltage of 200 kV. Temperature in situ experiments were carried out using a dual-axis TEM cryoanalytic holder Gatan model 636 compatible with the Temperature Controller model 900 SmartSet cold stage controller.

Foils for TEM were obtained by mechanical thinning with abrasives of various fineness, and further thinning was carried out electrochemically in an ethanol solution of 30% nitric acid at room temperature. The final thinning was carried out in an ion polishing unit with argon ions. The samples for the structural study were ultrathin TEM foils obtained by standard methods. Electron microscopy studies and foils preparation for TEM were carried out at the IRC Nanotechnology, Research Park, St. Petersburg State University, 199034 St. Petersburg, Russia.

## 3. Research Results and Discussion

### 3.1. Characteristic of Metamagnetostructural Transformation and Magnetocaloric Effect

**Figure 1:**
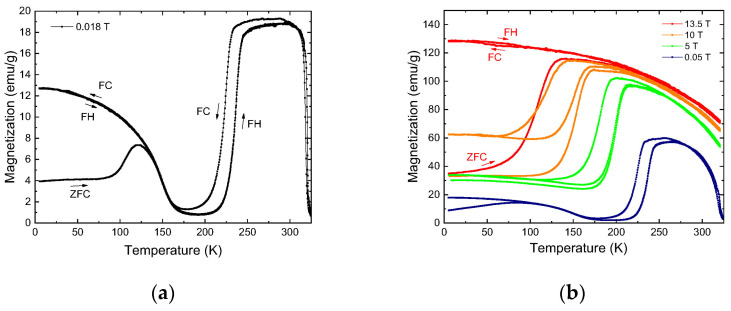

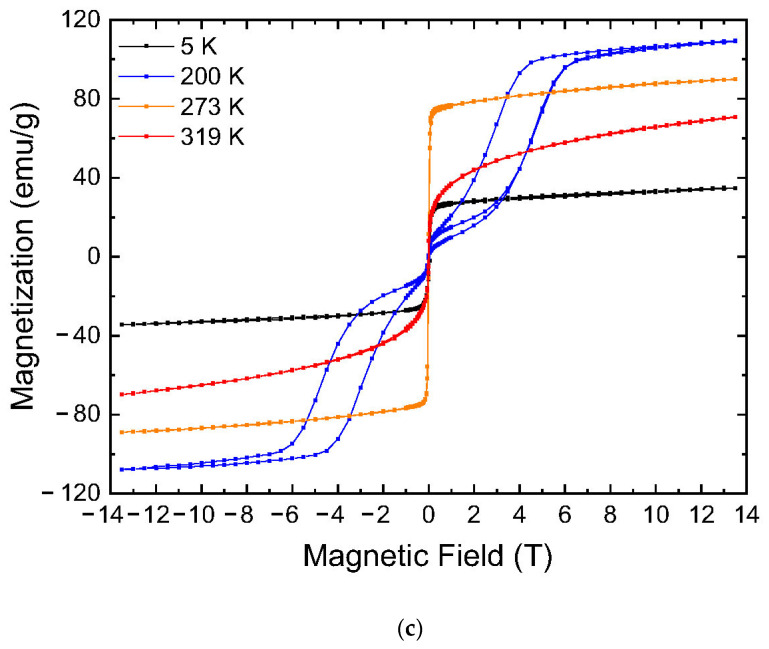
Graph showing the dependence of the sample’s magnetization (M) on temperature (T): (**a**)—in a field of 0.018 T; (**b**)—in fields of 0.05, 1, 3.5, 10, and 13.5 T; (**c**)—the dependence magnetization (M) on magnetic field (T) at temperatures 5, 200, 273, 319 K.

Using DSC (data present in [13]) methods and determining the temperature dependences of the magnetization (the results are present in Figure 1) in low and high fields, the start and finish temperatures of the direct (M*_s_* and M_f_) and reverse (A_s_ and A_f_) metamagnetostructural transformation and the Curie temperature (T_c_) were determined.

The magnetization data of the sample confirms the presence of a metamagnetostructural phase transformation. Study by magnetization in a field of 0.018 T: M_s_ = 230 K, M_f_ = 213 K, A_s_ = 226 K, A_f_ = 245 K, T_c_ = 323 K (Figure 1a).

The data of the isofield dependence of the magnetization show that the sensitivity of the material to the magnetic field was k_Ms_ = 8.9 K/T (Figure 1b), that is., an applied external magnetic field of 1 T, shifts the temperature of the metamagnetostructural transformation by 8.9 K toward lower temperatures. Isofield dependence 1–3 T in [13].

Isothermal curves of magnetization in magnetic fields up to ±13.5 T with an increase in temperature from 5 K, 200 K, 273 K, and 319 K are shown in Figure 1c. It can be seen here that, at a temperature of 5 K, in the region of magnetic frustration the martensitic phase behaves as an antiferromagnet, and the value of the magnetization corresponds to the value of the ZFC regime in isofield measurements (Figure 1b). At a temperature of 200 K, an induced magnetic field metamagnetostructural phase transition is observed. When a magnetic field is applied, ferromagnetic austenite begins to nucleate in the parent martensitic phase. With an increase in the magnetic field from 0 to 6.5 T, the entire martensite passes into the austenite phase, and then up to 13.5 T, the process of technical magnetization of ferromagnetic austenite occurs. When the magnetic field is removed at 4.6 T, on the contrary, a martensitic transition begins, when antiferromagnetic martensite appears in ferromagnetic austenite. It is worth noting that with a second increase in the magnetic field in the range from 0 to 4 T, the magnetization loop passes above the initial one. This is due to the fact that at 200 K, the previously induced austenite does not completely returns to the martensite phase. At a temperature of 273 K, ferromagnetic properties appear in the austenite region. At a temperature of 319 K near the Curie temperature, the dependence of the magnetization acquires a paramagnetic character.

**Figure 2:**
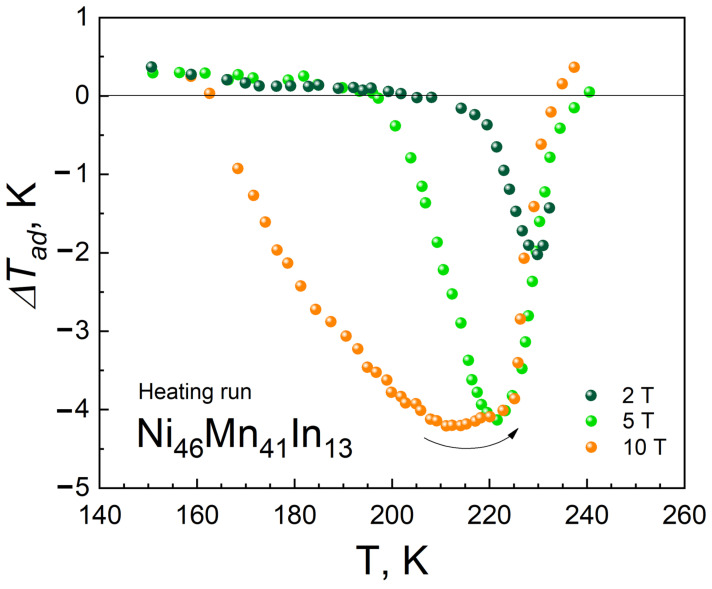
Temperature dependence of ∆T_ad_ obtained upon sequential heating sample in magnetic fields of 2, 5 and 10 T.

Figure 2 shows the dependences of the magnetocaloric effect ∆T_ad_ on temperature in magnetic fields of 2, 5, and 10 T. It can be seen that, in the temperature range below 160 K, a slight conventional magnetocaloric effect is observed in the region of magnetic frustration in the martensite phase. In the temperature range of 160–240 K, in the region of the metamagnetostructural phase transition martensite–austenite, an inverse magnetocaloric effect appears, and in the temperature range of the existence of ferromagnetic austenite, a positive increase in temperature, that is, a conventional magnetocaloric effect. It should be noted that an increase in the applied magnetic field causes a shift in the maximum values of the MCE to the low-temperature martensite phase. The obtained dependence is consistent with the data of direct measurements of the magnetocaloric effect in Heusler alloys of the Ni-Mn-In system [15,16,17,18,19,20,21,22,23]. Namely, in [24], the maximum value of the magnetocaloric effect was obtained equal to ∆T_ad_ = −5.5 K at a temperature T = 219 K in a magnetic field of 8 T, versus ∆T_ad_ = −4.2 K at a temperature T = 212 K in a magnetic field of 10 T obtained in our article. In a magnetic field of 5 T, the authors obtained a value of ∆T_ad_ = −4.8 K at a temperature of T = 224 K, against ∆T_ad_ = −4.1 K at a temperature of T = 222 K obtained by us.

The behavior of the temperature dependence ∆T_ad_(T) in the Ni_46_Mn_41_In_13_ compound under study in the region of the magnetic field-induced phase transition from antiferromagnetic martensite to ferromagnetic austenite and the presence of a large inverse MCE can be due to a significant difference in the values of magnetization M(T) in the martensitic phase compared to the austenitic phase. In turn, the difference in magnetization can be explained by the fact that in Mn-based Heusler alloys, magnetic moments are localized mainly on Mn atoms and the exchange interaction strongly depends on the Mn–Mn distance [25,26]. Therefore, any change in the configuration of the crystal lattice during the martensitic transition can change the strength of exchange interactions and, as a result, lead to a change in the magnetic properties of the alloy [27].

### 3.2. Structural Studies

According to the isofield dependences of the magnetization on temperature, it was found that the metamagnetostructural phase transition in this Ni_46_Mn_41_In_13_ bulk sample has a high sensitivity of characteristic temperatures to the magnetic field K_Ms_ = 8.9 K/T in fields of 1–3 T, as mentioned above, which is several times higher than the sensitivity for Heusler alloys of the Ni-Mn-Sb system and for magnetostructural phase transitions in the Ni-Mn-Ga systems. Therefore, it is expected that when the sample size is reduced to the size of thin films obtained from bulk samples, their sensitivity to an external magnetic field will not change (or even increase), and macro deformation, as a result of magnetically and thermally induced martensitic transition, will be large, which is extremely important for creating micro- and nanomanipulators, devices, and other products whose activation requires medium-power magnetic fields. (0.5–3 T, for example), and the value of the magnetocaloric effect will practically not change.

It is known that the thin-film state, in comparison with the bulk state, expands the temperature range of the austenitic phase for Ni-Mn-In systems [13]. Similar deviations in the behavior of the Ni-Mn-Ga system in the thin-film and bulk states were also observed for the Ni-Mn-Ga system [28,29]. However, information about the specific microstructures of various states of such systems (primarily with thermoelastic martensitic and metamagnetostructural transitions) is rather scarce, and this information is extremely necessary, both for applied purposes and for modeling functional materials.

**Figure 3:**
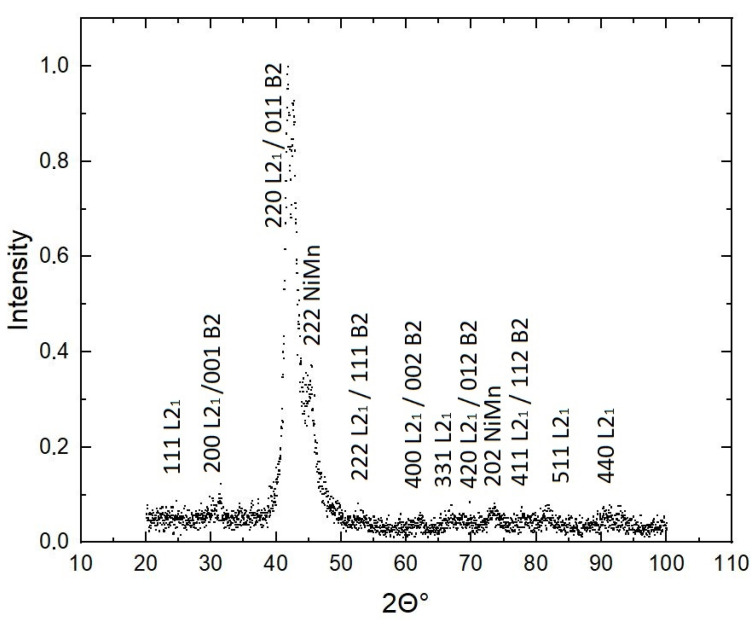
X-ray diffraction pattern of the Ni_46_Mn_41_In_13_ alloy at room temperature.

Figure 3 shows X-ray diffraction data for a Ni_46_Mn_41_In_13_ sample at room temperature (300 K), that is, above the martensitic transformation temperature of the alloy. It can be seen that all the main Bragg lines are «blurred», which indicates the non-single-phase nature and the presence of internal stresses. The peak in the diffraction pattern (111) is almost invisible, which indicates incomplete ordering. Characteristic diffraction lines (200) and (220) are both present, demonstrating the formation of B2-type ordering; a (331) peak associated with the emergence of the L2_1_ phase is also observed.

In order to unambiguously determine the structure in nonstoichiometric Heusler alloys from the point of view of «order–disorder», one can consider a stoichiometric (as simpler) compound in B2 and L2_1_ ordering types. The results of such a study are published in the article [30] for the Ni_2_MnAl alloy. The authors assume that, since the B2→L2_1_ transformation in Ni_2_MnAl occurs at a temperature at which diffusion proceeds quite slowly, the high-temperature B2 phase is retained at room temperature in a metastable state. This, in turn, prevents the exclusively formation of the thermodynamically more favorable phase L2_1_.

In addition, upon cooling, order–disorder transformations can overlap, and the alloy will tend to decompose and, as was shown in [11], transform into a two-phase composite L2_1_(Ni_50_Mn_25_X_25_) + L1_0_(Ni_50_Mn_50_). Determination of the effects of ordering and secondary nanoscale phases is very difficult from X-rays. In addition, at room temperature, there may be a small amount of the martensite phase, and some peaks can be attributed to the martensitic phase with low symmetry. Thus, X-ray diffraction data alone cannot unambiguously determine a small degree of disorder in an ordered structure or a low degree of order in a disordered structure [31]; therefore, a detailed analysis of structural studies by other methods is required.

**Figure 4:**
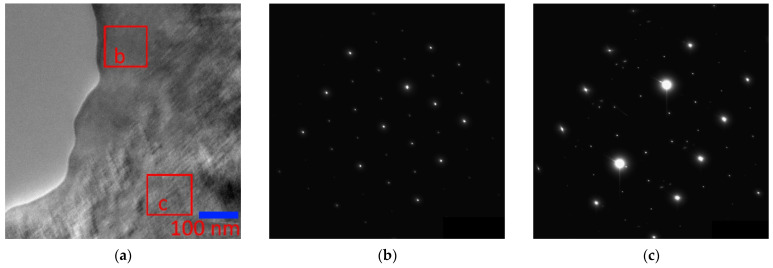
Micrograph of the Ni_46_Mn_41_In_13_ alloy (**a**) and microdiffraction patterns (**b**,**c**) from regions with different structures.

In connection with the foregoing analysis, we studied in detail the Ni_46_Mn_41_In_13_ alloy by transmission electron microscopy. The micrographs of the alloy in the austenitic state (Figure 4a) show both regions of the completely ordered L2_1_ phase (Figure 4b) and regions characterized by a tweed structure with diffuse scattering and additional reflections on the electron diffraction patterns (Figure 4c).

**Figure 5:**
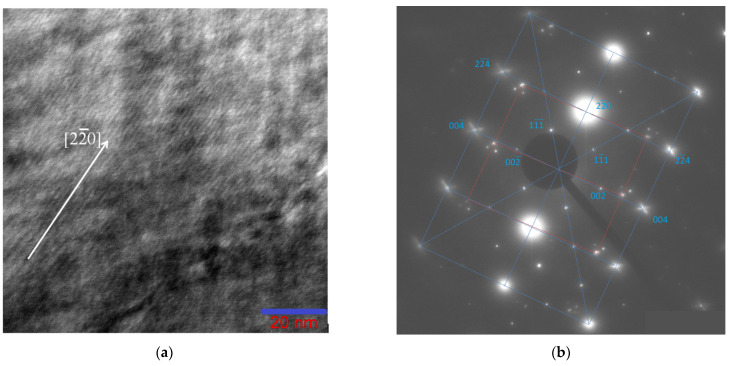
Bright-field (**a**) TEM image of the Ni_46_Mn_41_In_13_ alloy in the austenitic state at room temperature and the corresponding microelectron diffraction pattern (**b**) obtained from the entire area. The grating L2_1_ is shown in blue, with the [110] zone axis.

The electron diffraction patterns of the alloy in the austenitic state (Figure 5) showed consistently characteristic reflections of the B2 structure with a = 3.085 Å and superstructural reflections of the (111) plane, confirming the presence of the L2_1_ superstructure with a = 6.170 Å. In the microdiffraction pattern (Figure 5b) with [110]_L21/B2_ zone axis, both the main reflections B2 + L2_1_ with high intensity and the superstructural reflections B2 and L2_1_ with low intensity (the grating is highlighted in blue, indexes are for L2_1_) are present. Separately, it should be noted that not a single electron diffraction pattern revealed solely a B2 structure, so it may be concluded that the sample always contained ordered regions. In addition, a characteristic feature of this sample is that in all diffraction patterns of austenite, there are additional reflections localized by three, creating a grating (highlighted in red), one of the directions of which is parallel to [110]_L21_.

On the bright and dark field electron microscopy images of the austenite structure, a tweed contrast is observed with a distance between the stripes of ~10 nm (Figure 5a), and on the electron diffraction patterns near the main reflections of the L2_1_ phase (except for the superstructural (111)_L21_ and (001)_B2_) regions diffuse scattering in the form of two radial diffuse strands intersecting at an angle of ~115° along the directions [2$\bar{24}$]* and [2$\bar{2}$4]*. The tweed contrast bands are in accordance with the diffuse scattering bands: each system of contrast bands is orthogonal to one of the diffuse scattering directions. In addition to the tweed contrast, parallel very thin bands along <220>* with a distance of ~1.35 nm between them are observed (Figure 5a). This indicates that the grating contains micromodulations with a period of nine interplanar distances in the <004>* direction (d_004_ ≈ 0.15 nm).

The tweed contrast is observed in alloys and compounds in which the martensitic-type transformation is accompanied by pre-transition phenomena: the displacements of atoms towards the future phase. Displacements of atoms from equilibrium positions cause lattice deformations, which manifest themselves in the appearance of modulated structures, in particular, a tweed microstructure. Such tweed domains appearing in the pre-martensitic state probably serve as sites for martensite nucleation. In such a case, satellite reflections and characteristic diffuse scattering appear in the diffraction patterns. The tweed structure observed in this case is the result of the incorporation of the intermediate phase into the initial cubic phase. Such a structure in [32] is called not just pre-martensitic tweed, but an intermediate tweed phase, believing that the so-called pre-martensitic phase is actually an independent phase preceding the martensitic transformation.

**Figure 6:**
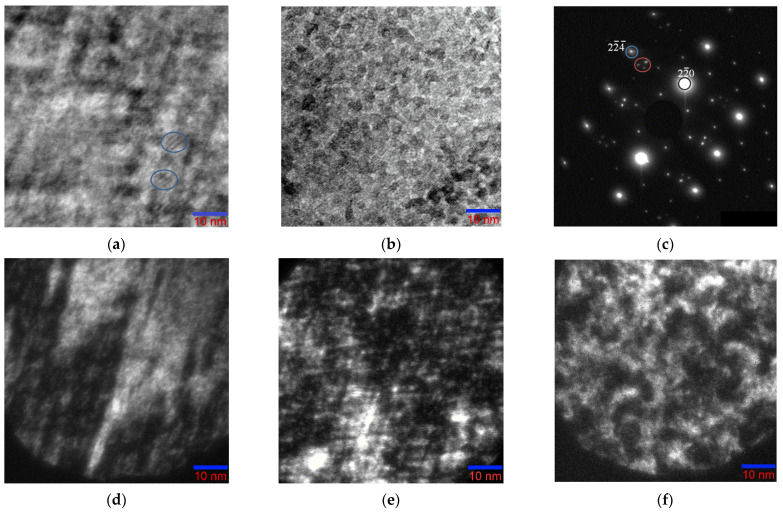
Structures of the Ni_46_Mn_41_In_13_ alloy in the austenitic state at room temperature: (**a**,**b**) bright-field TEM images; (**c**) microelectron diffraction pattern obtained from area (**a**); (**d**–**f**) dark-field images in reflections (2$\bar{24}$)_L21_, (2$\bar{2}$0)_L21_, and triple reflex, respectively. Zone axis [110]_L21_.

At moderate cooling rates, nonstoichiometric Ni_46_Mn_41_In_13_ can decompose into regions that differ both in chemical composition and levels of ordering, resulting in the formation of a modulated periodic structure. Such a structure develops as a result of self-organization of phases under the action of deformations, that is, displacements of atoms from equilibrium positions towards the future phase. The results obtained suggest that the phase transformation begins with the formation of local regions of L2_1_ oriented along directions that create a tweed contrast (orthogonal to the directions of diffuse strands along [2$\bar{24}$]* and [2$\bar{2}$4]*). Figure 6a clearly shows local moiré patterns (circled) arising from fully or partially coherent regions of the ordered phase ~5 nm in size. In some areas of the foil, almost square L2_1_ structural nanodomains ~3–5 nm in size, aligned along the directions of the tweed, are observed (Figure 6b).

Dark-field images obtained from the region in Figure 6a in the main lattice reflections of the L2_1_ phase, which have diffused scattering strands, correspond to one (Figure 6d) or two (Figure 6e) sets of tweed contrast bands. The dark-field image in the triple reflection, which we associate with decay products, is a classical modulated structure resulting from stratification by the spinodal mechanism [33] (Figure 6f). The triplet of reflections in electron diffraction patterns (Figure 4c and Figure 5b) is made of the main reflection and two extra reflections, the origin of which is associated with “punctures” of the Ewald sphere by diffuse strands that do not belong to the given section of the reciprocal lattice. Intensity bands located obliquely to the diffraction plane are depicted on the electron diffraction patterns as satellites near the Bragg reflections.

**Figure 7:**
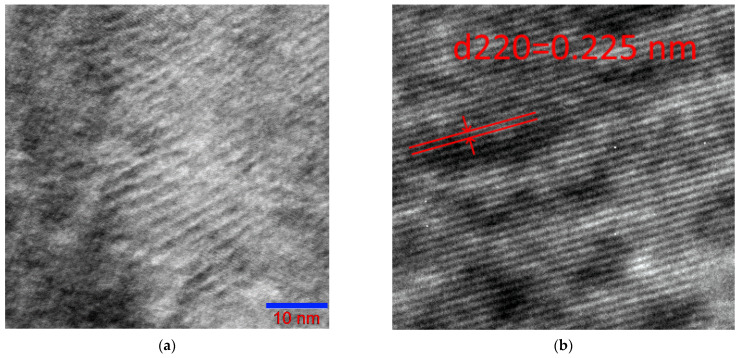
High resolution photomicrographs from the tweed texture area of the Ni_46_Mn_41_In_13_ alloy in the austenitic state at room temperature (**a**) and selected enlarged fragment of the photo (**b**).

High resolution TEM analysis revealed the microstructure of the tweed with alternating dark and light domains of 3–5 nm (Figure 7). Striped contrast extending across the entire field of Figure 7b, is formed by the projections of the {220}L2_1_ planes with the interplanar spacing. These bands run continuously through all domains, indicating that the boundaries between domains are coherent.

**Figure 8:**
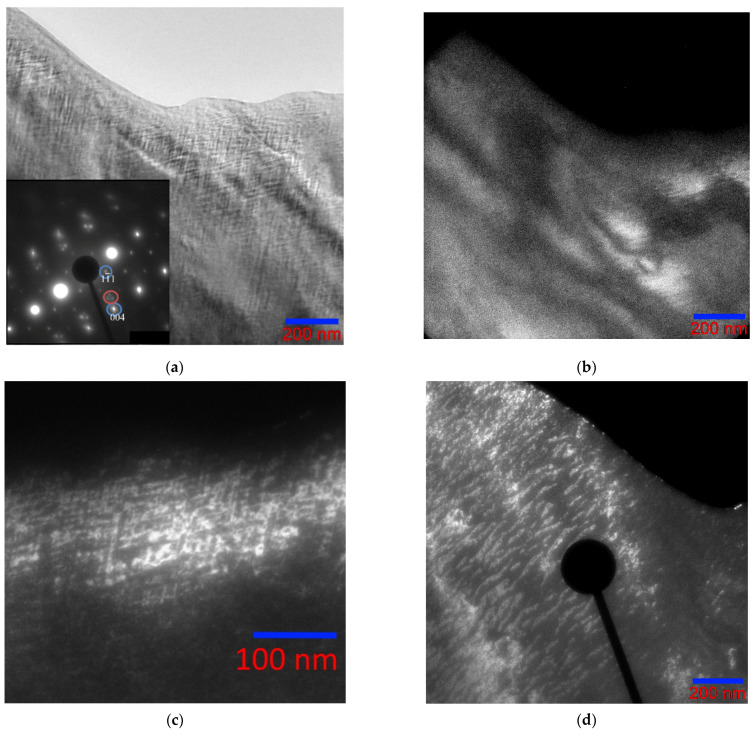
Bright-field (**a**) and dark-field (**b**–**d**) TEM images of the structure of the Ni_46_Mn_41_In_13_ alloy in the austenitic state and the corresponding microelectron diffraction pattern on the inset, obtained from the entire region (**a**), zone axis [110]_L21_, (**b**) in reflection (1$\bar{1}$1)_L21_, (**c**) in the reflection (004)_L21_, (**d**) in the triplet of reflections).

**Figure 9:**
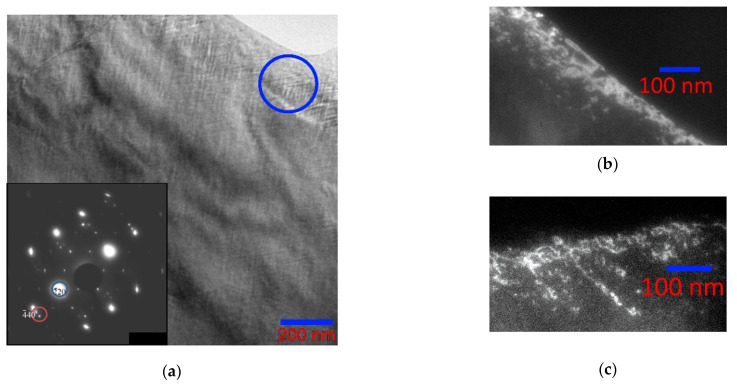
Bright-field image of the structure of the Ni_46_Mn_41_In_13_ alloy (**a**) (inset: SEAD electron diffraction pattern from the region in the upper right corner (tweed contrast) with the zone axis [110]_L21_ and dark-field images in reflections ($\bar{2}$20)_L21_ (**b**) and ($\bar{4}$40)_L21_ (**c**).

It should be recalled that the structure of non-stoichiometric compositions of Heusler alloys is metastable, even small inhomogeneities in composition or temperature changes can lead to the formation of various microstructures. For example, depending on the degree of atomic ordering, the Ni_46_Mn_41_In_13_ compound is expected to contain structural domains and antiphase boundaries (APBs) formed during the transition from the partially ordered B2 phase to the ordered L2_1_. In the next part of the work, we will analyze dark-field images obtained of both superstructural and basic reflections of the L2_1_ phase (Figure 8 and Figure 9). In the dark-field image (Figure 8b) obtained of the (1$\bar{1}$1)_L21_ superstructural reflection, bright regions correspond to zones with a large volume fraction of the L2_1_ phase, whereas dark regions correspond to B2-type ordered zones. It must be considered that some areas of L2_1_ and B2 may intersect at different heights and give an image with different gray intensities depending on the ratio L2_1_/B2 [34]. Since the ordering process in this compound is accompanied by decomposition, this explains why pronounced APBs are not observed in the superstructural reflection of the ordered phase, which will be less energetically favorable compared to the separation-matrix interfaces.

If a dark-field image is obtained in the (004)_L21_ reflection (Figure 8c), that is, in a reflection with diffuse scattering strands, then a contrast is observed from the APB separating the tweed domains. The dark-field image obtained in the triplet of reflections is shown in Figure 8d. The bright areas in the micrograph are periodic, evenly distributed curved strips in the form of “worms” 100–200 nm long. One can define this structure as a visualization of alternating domains of a partially ordered B2 phase and an ordered L2_1_ phase. Figure 9 shows dark-field APB images obtained in various reflections from a region characterized by a tweed structure (the region is circled in blue in Figure 9a).

Evidence that antiphase boundaries are magnetic domain wall stoppers was presented for the Ni_50_Mn_25_Al_12.5_Ga_12.5_ alloy (L2_1_ phase) in [35]. It was shown that the antiphase boundaries observed by the dark field method coincided almost perfectly with the magnetic domain walls detected using Lorentz microscopy. Zuo et al. [36] demonstrated periodic stripe magnetic domains in Ni_50_Mn_35_In_15_ observed using in situ Lorentz transmission electron microscopy, which are indeed very similar to the domains in Figure 8d.

© structure in the form of “worms” is similar to the classical modulated structure that occurs during concentration separation, resulting from stress fields, indicating local lattice distortions [37]. Moreover, the X-ray diffraction pattern of the sample (Figure 3) suggests the possibility of the presence of two phases of different composition, and structural metastability, as already mentioned, is an inherent property of Heusler alloys. The foregoing makes it possible to consider the coexistence of order–disorder in these alloys in unity with structural instability.

**Figure 10:**
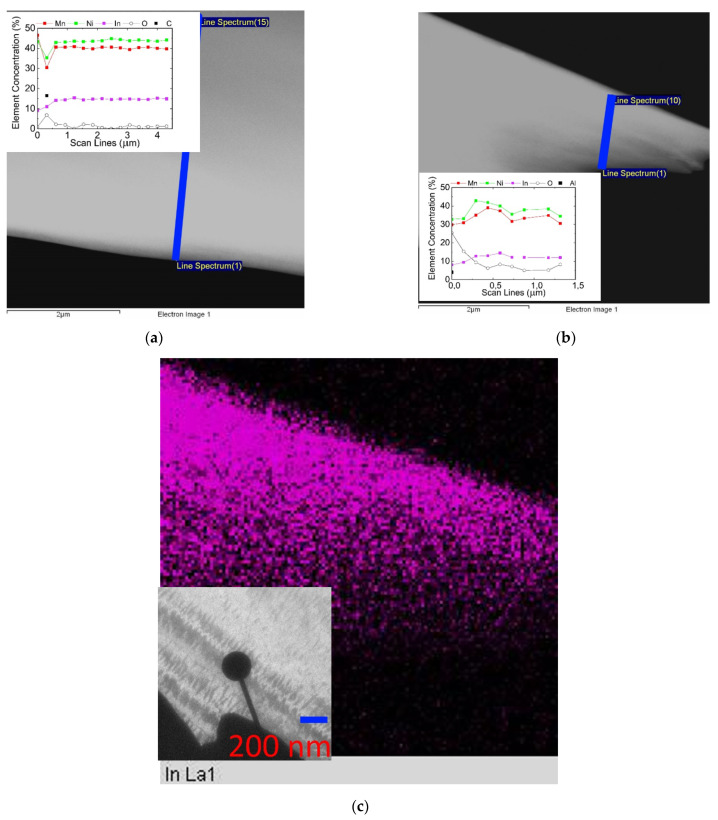
STEM images of local content of the elements O, In, Mn, Ni (on the graphs from bottom to top) along the scanning line with a large step (**a**) and a smaller step (**b**). The arrows indicate the scan lines. Distribution of indium (**c**) in the area circled in (**b**).

Next, for the alloy under study, the distribution of the elements Mn, Ni, and In was obtained, and the local concentration of these elements along the scanning line was determined. Scanning from the edge into the depth of the sample with a step of ~300 nm was performed as shown in Figure 10a. The distribution of the elements (see inset), with the exception of the very edge of the foil, did not reveal fluctuations in the composition throughout the entire scanning range. Scanning with a step of ~150 nm, commensurate with the size of the worm-shaped strips (in the inset at the bottom of Figure 10c), revealed a slight fluctuation in the composition of both Ni, Mn, and In (in the inset at the top of Figure 10b). This indicates that the transition to a two-phase composite in this alloy does not occur according to the scenario proposed in [11]. However, this does not rule out the presence of nanoscale regions resulting from separation by the spinodal mechanism into regions with different contents of indium, manganese, or nickel. The alloy Ni_46_Mn_41_In_13_ exhibits a phase decomposition tendency, even without additional low temperature annealing, only in the process of cooling with a furnace provided a suitable system for studying the early stage of decomposition. Microstructural observations indicate that the decay mechanism is indeed spinodal rather than nucleation and growth. The TEM method observes structures that correspond to nanoscale decay uniformly distributed inside the grain. Moreover, since the decay is considered not only as a chemical stratification, but also from the point of view of ordering, in this case we can speak more about conditional spinodal decomposition, rather than about classical spinodal decomposition. In addition, the diversity of structures indicates a high defectiveness, which hinders the formation of a full-fledged phase transformation of the martensitic type, which is typical not only for Heusler alloys [38].

### 3.3. Austenite–Martensite Boundary

**Figure 11:**
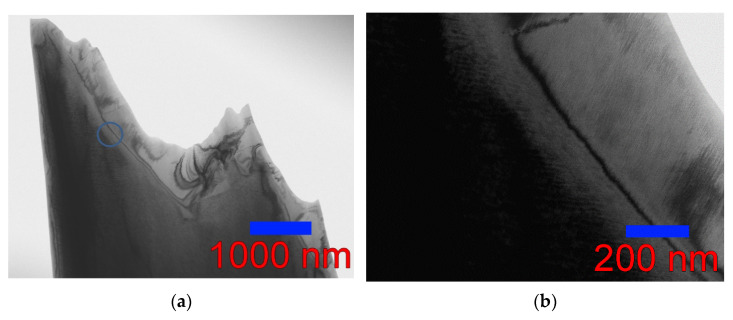

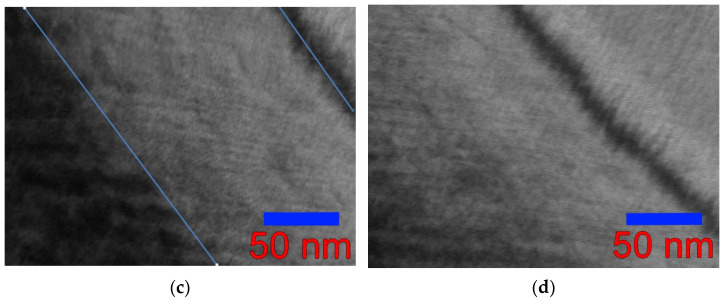
A–M boundary (100 K) at different magnifications (upper right corner—austenite, lower left corner—martensite), bright-field images.

The features of the fine structure of the wedge-shaped foil of the Ni_46_Mn_41_In_13_ alloy observed in this work showed that the martensitic transformation is blocked at a distance of about 600 nm from the edge of the sample at a plate thickness of less than 50 nm, where the formation of a martensitic structure does not occur even at liquid nitrogen temperature [13]. This size effect, shown in Figure 11a provided us with the opportunity to observe the austenite–martensite interface in the Ni_46_Mn_41_In_13_ alloy (Figure 11b,c). The boundary is not sharp and is a fairly wide transition layer ~150 nm wide. Such blurring indicates a high level of stresses, especially on the austenite side (Figure 11d). It can be seen how the banded contrast changes going from austenite (upper right corner) to martensite (lower left corner) through the transition layer. Of the two directions of the thin banded austenite contrast, only one direction remains with the same periodicity cane be seen at first, and then the distance between the bands increases, and a coarse black-and-white banded contrast is observed on the martensite side.

**Figure 12:**
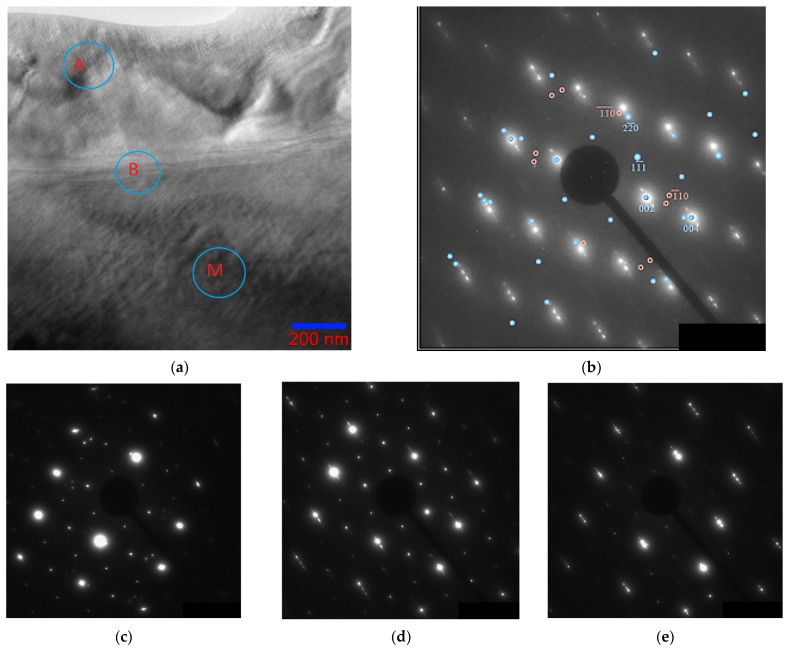
Structures of the Ni_46_Mn_41_In_13_ alloy at the A–M interface at 215 K: bright-field image (**a**), combined austenite–martensite electron diffraction pattern (**b**), electron diffraction patterns from the austenite region (**c**), A–M (“B” in circle) boundary (**d**) and martensite (**e**). Zone axis [110]_L21_.

Electron diffraction patterns belonging to the austenite region, the martensite region, and directly to the interface were obtained (indicated in Figure 12a). Figure 12b brings together two sets of reflections; reflections from the electron diffraction pattern of austenite with zone axis [110]L2_1_ are superimposed on the electron diffraction pattern obtained from the martensite region. It can be seen from the combined austenite–martensite diffraction patterns that, as the transformation progresses, all reflections of the L2_1_ phase (reflections of the (220), (002), (004), and (224) types), except for the superstructural ones (111), split in the direction of one of the two diffuse scattering strands near reflections of the (004), (224), and (444) types. In this case, the corresponding planes of the L2_1_ phase transform into the planes of martensite. The appearance of extra reflections on the electron diffraction patterns, at distances that are ^1^/_7_ of the distance between the main structural reflections, characterizes the martensitic phase as a modulated 14 M, and the martensitic transformation occurs according to the L2_1_ → 14 M type, which is consistent with the findings of other authors [39,40,41]. Banding in bright-field images of martensite is perpendicular to the direction of the splitting of reflections in the electron diffraction patterns. For reflections belonging to the austenite phase, ordered according to type B2 (in Figure 12b, these reflections are of the (110) and (020) types with two satellites circled in red). When approaching the austenite–martensite interface, one of the three reflections first disappears, then they all vanish. This suggests that these reflections still belong to a phase with a different type of ordering, and not to secondary phases or oxides of manganese and nickel.

**Figure 13:**
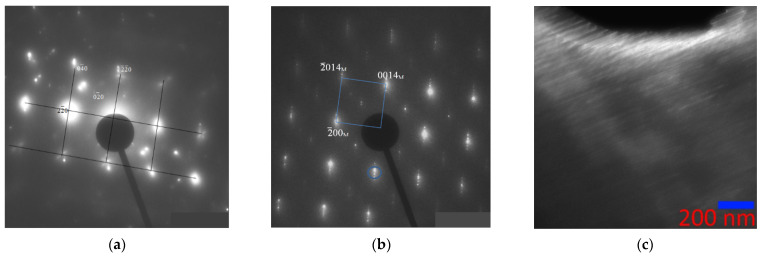
Structure of the Ni_46_Mn_41_In_13_ alloy at 208 K: (**a**)—electron diffraction pattern of the austenite structure, zone axis [001]_L21_; (**b**)—electron diffraction pattern of the martensite structure, zone axis [0$\bar{1}$0]_14M_; (**c**)—fine structure of martensite, dark-field image in the (00$\bar{14}$)M reflection (circled).

Figure 13a shows the electron diffraction pattern of the austenite structure with zone axis [001]_L21_ and the electron diffraction pattern of the martensite structure, which has a modulated lattice with a periodicity of seven planes along the [$\bar{11}$0]L2_1_ direction (Figure 13b), corresponding to 14 M. In this case, the martensite structure is in the form of plates with an internal streaky structure (Figure 13c). Banding in dark-field images of martensite is perpendicular to the direction of splitting of reflections in electron diffraction patterns.

The appearance of such a variety of structures obviously suggests that the crystal structure of the martensite phase will strongly depend on the degree of ordering of the austenite phase. In addition, the coexistence of regions L2_1_ and B2 in austenite apparently leads to the impossibility of complete martensitic transformation in the entire volume of the material, since the propagation of martensitic transformation is hindered in a medium inhomogeneity in the microstructure.

## 4. Summary

The Ni_46_Mn_41_In_13_ Heusler alloy was studied by the measurement of the dependence of magnetization on temperature in magnetic fields up to 13.5 T. The dependence of magnetization showed a shift of the characteristic temperatures in magnetic field of K_Ms_ = 8.9 K/T. With a further increase in the magnetic field, the hysteresis expands, and in a field of 13.5 T, blocking of the transformation is observed, and only the austenite (strongly magnetic phase) is observed in the structure. The magnetocaloric effect, measured by the direct method in the temperature range of 140–240 K and magnetic fields of 2, 5, and 10 T, demonstrated the maximum value in a magnetic field of 10 T of the inverse effect −4.2 K in the region of martensitic transformation.

The structure of the non-stoichiometric Ni_46_Mn_41_In_13_ Heusler alloy was studied by TEM methods in the temperature range of 353–215 K. At least two processes were established; one is where the cubic parent phase undergoes shear transformation in combination with diffusion, creating a tweed contrast consisting of a coherent mixture of the parent composition and L2_1_ ordered domains. The second process is associated with stratification into two phases that differ in composition and ordering. Both processes are a single ordering and decay phenomenon in which two phase transformations occur simultaneously, namely, atomic ordering and solid solution decomposition. It is not determined which of these transformations is the leading one. Both processes coexist and occur under the influence of each other. This makes it possible to consider the coexistence of order–disorder in these alloys in unity with structural instability and martensitic transformation.

A qualitative analogy was observed between the effects of an increase in the external magnetic field and a decrease in the thickness of the alloy sample on the manifestation of the martensitic transition in the alloy. Both factors lead to the same qualitative result: a decrease in temperature and subsequent complete blocking of the martensitic phase transition.

The results obtained are considered a single phenomenon in which two processes occur simultaneously, namely, atomic ordering and solid solution decomposition. As a result, a structure is formed consisting of ordered domains separated by decay particles. Such a process occurs, for example, in some alloys of the gold–copper–silver system [42] and is a complex continuous “ordering-decomposition” reaction. Both processes coexist and occur under the influence of each other. This makes it possible to consider the coexistence of order–disorder in these alloys in unity with structural instability and martensitic transformation. The results of this study indicate that the concentration stratification occurs by the mechanism of spinodal decomposition (conditionally spinodal decomposition) into nanoscale regions.

## Data Availability

Not applicable.
